# Substrate Dependence of CdSe/ZnS Quantum-Dot Light-Emitting Diodes: A Comparative Study between Rigid Glass and Flexible Plastic Substrates

**DOI:** 10.3390/nano13111780

**Published:** 2023-05-31

**Authors:** Seyoung Lee, Jimyoung Kim, Honyeon Lee

**Affiliations:** Department of Electronic Materials, Devices and Equipment Engineering, Soonchunhyang University, Asan 31538, Republic of Korea; schworld21@sch.ac.kr (S.L.); wlaud604@naver.com (J.K.)

**Keywords:** quantum dots, CdSe/ZnS, polyethylene naphthalate (PEN), glass, transient electroluminescence

## Abstract

The purpose of this study was to investigate the effect of substrate characteristics on the performance of quantum-dot light-emitting diodes (QLEDs) with the aim of developing high-performance flexible QLEDs. Specifically, we compared QLEDs made with a flexible polyethylene naphthalate (PEN) substrate to those made with a rigid glass substrate, using the same materials and structure except for the substrates. Our findings indicate that the PEN QLED had a 3.3 nm wider full width at half maximum and a 6 nm redshifted spectrum in comparison to the glass QLED. Additionally, the PEN QLED exhibited a 6% higher current efficiency, a flatter current efficiency curve, and a 2.25-V lower turn-on voltage, indicating superior overall characteristics. We attribute the spectral difference to the optical properties of the PEN substrate, such as light transmittance and refractive index. Our study also revealed that the electro-optical properties of the QLEDs were consistent with the electron-only device and transient electroluminescence results, which suggests that the improved charge injection properties of the PEN QLED were responsible. Overall, our study provides valuable insights into the relationship between substrate characteristics and QLED performance, which can be used to develop high-performance QLEDs.

## 1. Introduction

Research on developing high-performance CdSe quantum-dot light-emitting diodes (QLEDs) has been ongoing since the first electroluminescence observation from CdSe quantum dots [1]. The initial devices suffered from low efficiency and poor stability, but a breakthrough was achieved with the development of CdSe/ZnS core–shell quantum dots [2,3,4,5]. Subsequent research focused on improving the efficiency and stability of CdSe/ZnS QLEDs, resulting in the development of novel device architectures [3,6,7,8,9]. These advancements have led to highly efficient and stable CdSe/ZnS QLEDs with tunable emission colors and narrow linewidths, making them a promising technology for electronic display applications.

As the use of electronic displays expands, the importance of flexible displays is growing. Therefore, the development of flexible QLEDs is crucial, and various research has been conducted on this topic [10,11,12,13]. To achieve flexible displays, flexible substrates, such as polymer plastics [14,15] or thin metal plates [16,17], must be utilized. However, metal plates face challenges, including limited-bending resilience, the formation of parasitic capacitance with display electrode lines, and excessive weight, making their practical implementation difficult. On the other hand, plastic substrates can be made thin and lightweight while exhibiting excellent bending resilience, making them the preferred choice for flexible displays.

In traditional rigid displays, indium tin oxide (ITO) has commonly been used as the transparent electrode material. However, ITO is unsuitable for flexible displays due to its susceptibility to damage from bending. Consequently, research has explored the use of thin metals with sufficient transparency as transparent electrodes. However, achieving low electrical resistance with thin metal electrodes often results in excessively low optical transparency, posing challenges for their application as transparent electrodes. To overcome these challenges, a transparent electrode structure based on a dielectric/metal/dielectric stack has been proposed [18,19]. This structure enables the attainment of optical transparency and electrical conductance comparable to ITO, serving as a viable alternative. Electrodes utilizing oxide/metal/oxide (OMO) structures, with oxides as dielectric materials, offer desirable optical and electrical properties, as well as ease of fabrication, making them suitable as transparent electrodes for flexible displays.

In this context, we fabricated CdSe/ZnS QLEDs using OMO [10,18,19,20] electrodes on both glass and plastic substrates, and analyzed their characteristics. Most reports have emphasized the similarity of the properties of flexible QLEDs to those of rigid QLEDs, but it is reasonable to expect that changes in the materials of the substrate and electrodes will cause differences in the electro-optical properties of QLEDs. Our study investigates the impact of substrate characteristics on the performance of QLEDs and interprets the causes of differences in the properties of the devices. We report the results of our work, including transient electroluminescence (EL) [21,22,23] data, energy-band parameters, the light transmittance of each substrate, and the electro-optic properties of QLEDs.

Although CdSe/ZnS QLEDs exhibit superior electro-optic properties, the environmental toxicity associated with Cd presents a significant obstacle to their commercialization. Consequently, extensive efforts have been made to develop Cd-free quantum-dot (QD) materials in response to environmental concerns. However, our focus is not on the properties of QD materials themselves but rather on investigating the dependence of QLED characteristics on different substrate types. In this regard, CdSe/ZnS QLEDs serve as valuable test vehicles for our study, due to their simple fabrication process and reliable device properties.

## 2. Experimental Methods

**Figure 1:**
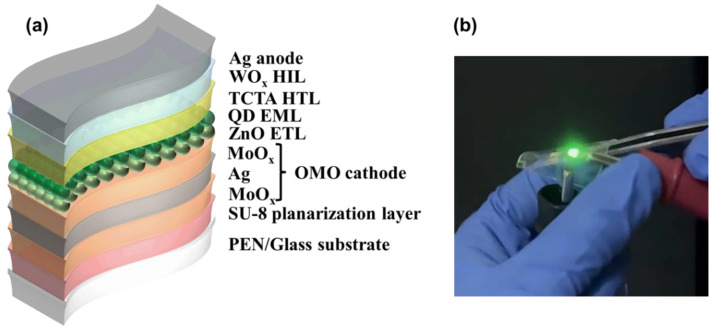
(**a**) Schematic showing the stacking structure of fabricated quantum-dot light-emitting diode (QLED), and (**b**) an image of the fabricated QLED emitting light.

Glass substrates with a thickness of 500 µm and polyethylene naphthalate (PEN) substrates [24,25,26] with a thickness of 100 µm were used for the devices. Each substrate had dimensions of 25 mm × 25 mm, and four QLED devices were fabricated on each substrate using identical conditions. The dimensions of each QLED device were 2.0 mm × 2.0 mm. The glass substrate is a common alkali-free glass used for the fabrication of display devices, while PEN is a substrate often used for fabricating prototype flexible displays due to its excellent thermal stability and visible light transmittance. To achieve a flat surface on the PEN substrate, a 2-µm thick planarization layer was created using SU-8 2002 [27], an epoxy-based photoresist. To ensure that the conditions for comparing the characteristics of the devices made of glass and PEN were the same, the same planarization layer was also produced on the glass substrate. After the planarization layer was fabricated, both substrates were subjected to a 300-s O_2_ plasma treatment using oxygen gas at a gas flow rate of 30 standard cubic centimeters per minute, under a pressure of 10^−4^ Torr, and with an RF power of 100 W. This treatment enhanced the adhesion of the OMO electrodes that were deposited on the upper surface of the planarization layer, thereby improving the overall reliability of the devices. For the OMO transparent bottom cathode, a 10 nm MoO_x_/12 nm Ag/10 nm MoO_x_ stacking structure was deposited using vacuum thermal evaporation after the O_2_ plasma treatment. This stacking structure was selected based on prior research [10]. The MoO_x_ layers were deposited using 99.99% pure MoO_3_ powder, while the Ag layer was deposited using 99.99% pure Ag granules. The deposition rate was 1.0 × 10^−1^ nm/s for both of Ag and MoO_x_. A layer of zinc oxide (ZnO) nanoparticles, with a thickness of 20 nm, was spin-coated onto the OMO cathode to create an electron transporting layer (ETL). The ZnO nanoparticles were synthesized in-house using the sol-gel method [24], and were then dispersed in ethanol to form a solution with a concentration of 32.9 mg/mL [28]. A green-emitting, quantum-dot (QD) emission layer (EML), with a thickness of 12 nm, was then spin-coated onto the ZnO ETL. The QD solution was prepared by dispersing CdSe/ZnS core–shell QD particles purchased from Global ZEUS (Korea) in heptane at a concentration of 5.0 mg/mL. Next, the hole transporting layer (HTL) and hole injection layer (HIL) were deposited sequentially on top of the QD EML using 99.8% pure 4,4′,4-Tris(carbazole-9-yl)triphenylamine (TCTA) purchased from LumTec Corp. (Taiwan) and 99.99% pure WOx as source materials via vacuum thermal evaporation. A 100 nm thick Ag anode was deposited on the HIL using vacuum thermal evaporation. The deposition rates for the HTL, HIL, and anode were 5.0 × 10^−2^ nm/s, 1.0 × 10^−1^ nm/s, 1.2 × 10^−1^ nm/s, respectively. Metal shadow masks were used for all patterning in the device fabrication process, without the use of photolithography. The thickness of spin-coated layers, the ETL and EML, was measured using an XE7 atomic force microscope (Park Systems Inc., Suwon, Republic of Korea). Based on these processes, bottom emission QLEDs were fabricated, and Figure 1 depicts the structure diagram, as well as an image of the fabricated QLED emitting light.

**Figure 2:**
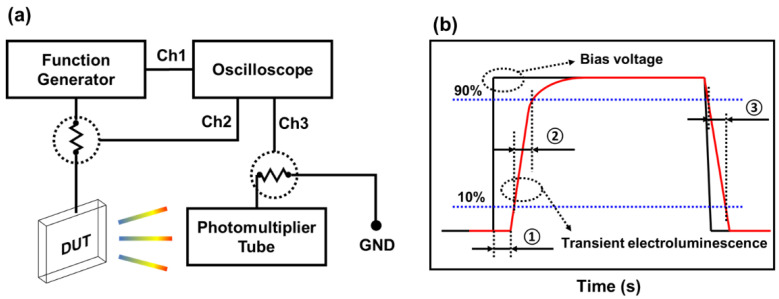
Diagram (**a**) illustrates the schematic of the transient electroluminescence measurement system, which includes a function generator, photomultiplier tube, and oscilloscope. The device under test (DUT) and ground (GND) are also indicated. Diagram (**b**) shows the definitions of delay time, rise time, and fall time, which are indicated by ①, ② and ③, respectively.

The current-voltage-luminance (I-V-L) characteristics of the fabricated QLEDs were measured using an I-V-L tester, which consisted of a Polaronix M6100 power supply (McScience Inc., Suwon, Republic of Korea) and a SpectraScan PR-650 spectrophotometer (JADAK Inc., North Syracuse, NY, USA). To analyze the charge transport and luminescence characteristics inside the device, we analyzed the transient EL characteristics using a self-built transient EL measurement system equipped with a function generator, photomultiplier tube, and oscilloscope, as shown in Figure 2a. To measure the transient EL, we used a square wave with a frequency of 1 kHz and a duty ratio of 50%. The applied voltage waveform and the measured EL waveform are shown in Figure 2b. Here, the delay time is the time difference from the application of the voltage to the occurrence of the EL signal, the rise time is the time taken for the EL signal to increase from 10% to 90% of the saturation value, and the fall time is the time taken for the EL signal to decrease from 90% to 10% of the saturation value. To measure the energy-band parameters and light transmittance of each layer of QLEDs, we used ultraviolet photoelectron spectroscopy (UPS) and ultraviolet-visible spectroscopy.

## 3. Results and Discussion

**Figure 3:**
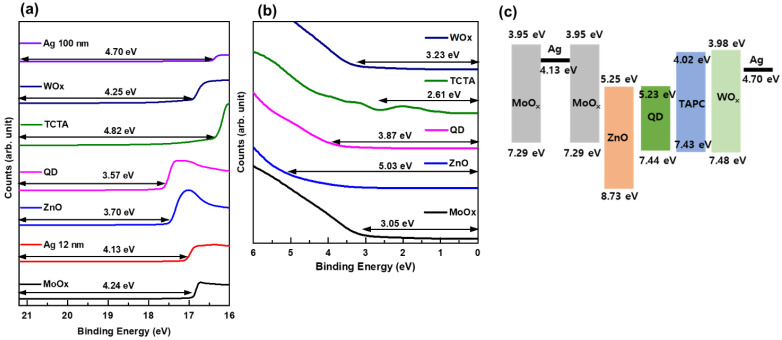
Ultraviolet photoelectron spectroscopy results; (**a**) secondary electron cut-off spectra and (**b**) Fermi edge regions, and (**c**) the QLED energy-band diagram.

Each layer’s energy band in QLED was obtained by combining the data from UPS and Tauc plot [29]. The cutoff energy of the secondary electron obtained from the UPS data is shown in Figure 3a, and the highest occupied molecular orbital (HOMO) energy level is shown in Figure 3b. Films for UPS measurements were deposited on ITO-coated glass substrates. The ITO film was used to align the Fermi level in ZnO, QD, TCTA, MoOx, and WOx films, as well as to prevent charge accumulation. A He I line with an energy of 21.2 eV was employed as the UV light source for UPS measurements. The work function of each sample material was determined by subtracting the secondary electron cut-off energy (Figure 3a) from the 21.2 eV He I line energy. The calculated work function values are indicated on the spectra for each material in the figure. From the results shown in Figure 3b, the HOMO values of each material can be determined relative to the Fermi level. These values are also displayed on the spectra. By combining the results from Figure 3a,b with the bandgap energy obtained from the Tauc plot, an energy band diagram is obtained (Figure 3c). Figure 3c is a diagram of the energy band levels inside the QLED device, extracted from the obtained data. Compared to electron injection into QDs, it can be observed that the barrier for hole injection is high, which can result in electron excess and hole deficiency, and consequently affect the device characteristics.

**Figure 4:**
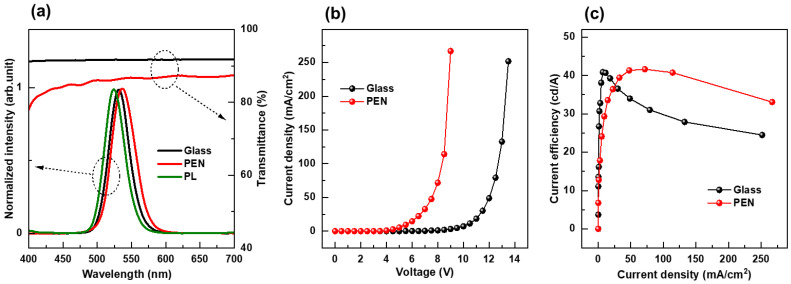
Optical and electrical properties of QLEDs fabricated on PEN and glass substrates: (**a**) Visible light transmittance curves of PEN and glass substrates, electroluminescence spectra of QLEDs, and a QD photoluminescence spectrum; (**b**) Current density-bias voltage curves of QLEDs; and (**c**) Current efficiency-current density curves of QLEDs.

Figure 4a displays the visible light transmittance curves of both glass and PEN substrates, as well as the EL spectra of the QLEDs fabricated using these substrates. The figure also includes the QD photoluminescence (PL) spectrum for comparison. Both substrates had good transmittance, with glass exhibiting a very flat transmittance curve while PEN had a slightly lower transmittance, and its curve decreased as the wavelength became shorter. The EL spectrum of the QLED fabricated using PEN substrate was redshifted by 6 nm when compared to the EL spectrum of the QLED fabricated using glass substrate. This redshift was caused by the higher transmittance of the longer wavelengths in the PEN substrate’s transmittance curve compared to the shorter wavelengths. Furthermore, the full width at half maximum (FWHM) of the EL spectrum for the PEN QLED was 3.3 nm wider, compared to that of the glass QLED. This is because the refractive index of PEN is around 1.7 [30], which is higher than the glass refractive index of 1.5, resulting in a weaker microcavity effect inside the QLED device. This widens the FWHM of the emission spectra.

Regarding QLEDs using glass and PEN substrates, the current density-voltage curves (Figure 4b) and current efficiency-current density curves (Figure 4c) are shown. The current efficiency is calculated using the formula: current efficiency = luminance/current density. Although not depicted in the figure, the luminance turn-on voltage—defined as the voltage at 1 cd/m^2^—was 3.30 V for the PEN QLED and 5.55 V for the glass QLED. This confirms that using a PEN substrate lowers the voltage required for emission to start. The maximum efficiency observed in the current efficiency-current density curve was 43.3 cd/A for the PEN QLED and 40.9 cd/A for the glass QLED. This indicates that efficiency was higher when employing a PEN substrate, but the difference was not significant. However, the shape of the current efficiency-current density curve varied significantly. The glass QLED had the highest efficiency at exceptionally low current densities, but the efficiency decreased rapidly as the current density increased. In contrast, the PEN QLED exhibited the highest efficiency at a somewhat higher current density, and the decline in current efficiency was not as steep as that of the glass QLED.

**Figure 5:**
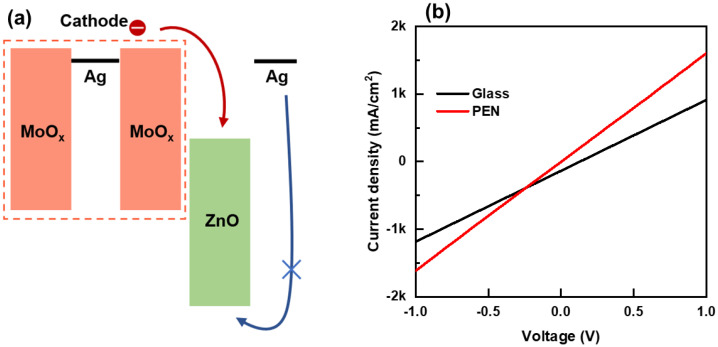
(**a**) The energy-band diagram of EODs on PEN and glass substrates, and (**b**) current density-bias voltage curves of EODs on PEN and glass substrates.

The differences observed in the current density-bias voltage and current efficiency-current density characteristics between QLED devices using PEN and glass substrates are presumed to be related to differences in their charge injection properties. To investigate this further, we fabricated electron-only devices (EODs) with the same stacking structure of OMO electrode/ZnO ETL/Ag electrode and analyzed their voltage-current characteristics. The energy-band diagram of the EOD is shown in Figure 5a. The bias voltage-current density curves of the EODs for both substrate types are presented in Figure 5b, clearly indicating that the electron injection into the EOD on the PEN substrate was higher compared to that on the glass substrate [6,29].

**Figure 6:**
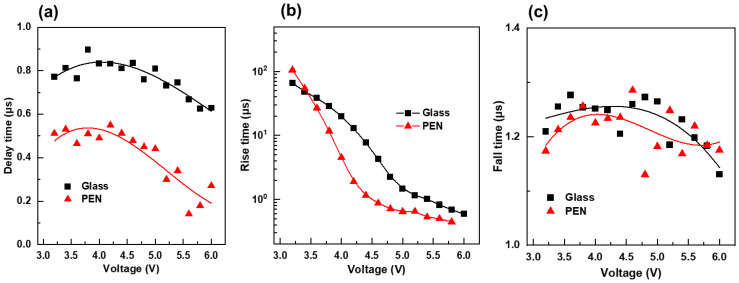
Transient electroluminescence results of PEN and glass QLEDs; (**a**) delay time, (**b**) rise time, and (**c**) fall time. Symbols represent the measured values from the transient electroluminescence curves. Solid lines in (**a**,**c**) indicate third order polynomial fit lines to the measured data.

Figure 6a–c show the results of transient EL measurements performed on PEN and glass QLEDs, which present the delay time, rise time, and fall time, respectively. The delay time of the glass QLED was significantly longer than that of the PEN QLED. The delay time is the time difference between the application of the bias voltage and the start of light emission, and the shorter delay time of the PEN QLED compared to the glass QLED indicates that charge injection is more efficient in the PEN QLED. Both the glass and PEN QLEDs exhibited a decrease in rise time with increasing bias voltage, but the slope of the decrease in the PEN QLED was larger than that of the glass QLED. The rise time represents the time it takes for charge distribution within the QLED device to stabilize [21]. An increase in the applied bias voltage leads to an increase in the flux of injected charges, resulting in a faster attainment of steady state. The faster decrease in rise time in the PEN QLED indicates that the process of charge injection and light emission occurs more effectively in the PEN QLED than in the glass QLED. Fall time was not affected by the magnitude of the applied bias voltage, and there was slight difference between the glass and PEN QLEDs. The fall time is the time taken for accumulated charges within the device to be recombined radiatively after the bias voltage is turned off, and it is less related to charge injection characteristics.

The results in Figure 4, Figure 5 and Figure 6 indicate that the PEN QLED had a lower turn-on voltage and a more stable, flat current efficiency-current density curve compared to the glass QLED. Additionally, the highest efficiency was slightly higher at 6% when using PEN. These findings are consistent with the EOD current-voltage curves and transient EL results, which suggest that charge injection is more effective in PEN QLED, and thus exhibits better electro-optical characteristics. Similar to the findings of this study, variations in device characteristics between glass and plastic substrates, even with identical structures, have been reported in flexible solar-cell research [31,32]. However, the exact cause of these differences has not been clearly identified. In this study, through EOD and transient EL experiments, it was found that differences in charge injection characteristics contribute to these device performance variations. However, the understanding of factors causing such differences in charge injection characteristics is still limited. Nevertheless, it is speculated that differences in surface temperature during the OMO electrode deposition process due to variations in thermal diffusivity and thickness between glass and plastic substrates could affect the oxidation state of Ag. Due to the susceptibility of Ag to oxidation, the presence of oxygen from MoO_x_ at the Ag/MoO_x_ interface could result in the formation of Ag-Mo-O oxide compounds [33]. This phenomenon may cause variations in the current flow from the cathode to the anode. However, further experimental research is necessary to find clear evidence for these speculations.

## 4. Conclusions

The study successfully fabricated flexible PEN QLEDs using an OMO electrode on a plastic PEN substrate, and compared their performance to rigid glass QLEDs. The results showed that the emission wavelength redshifted by 6 nm and the FWHM increased by 3.3 nm when using the PEN substrate due to its higher transmittance at longer wavelengths and higher refractive index. To accurately represent color, it is necessary to consider the optical properties of the flexible substrate in device design. The PEN QLED had superior electrical and optical properties with a 2.25-V lower turn-on voltage, a flatter current efficiency-current density curve, and a 6% higher current efficiency compared to the glass QLED. These variations are attributed to the differences in charge injection properties between the QLEDs on glass and PEN substrates. The variances in substrate thickness and thermal diffusivity could potentially lead to variations in surface temperature during the OMO deposition process, consequently affecting the Ag/MoO_x_ interfacial states and current flow properties. However, additional experimental studies are required to validate these speculations. The findings of this study will be useful in the design of flexible QLEDs with superior properties, including the substrate choice.

## Data Availability

Not applicable.
